# Responses to medical treatment in patients with metastatic unresectable small intestinal neuroendocrine tumors – A single center study of 378 patients

**DOI:** 10.1111/jne.70138

**Published:** 2026-02-18

**Authors:** Cecilie Slott, Seppo W. Langer, Peter Oturai, Stine Møller, Carsten Palnæs Hansen, Andreas Kjaer, Pernille Holmager, Marianne Klose, Rajendra Singh Garbyal, Ulrich Knigge, Mikkel Andreassen

**Affiliations:** ^1^ ENETS Center of Excellence Copenhagen University Hospital—Rigshospitalet Copenhagen Denmark; ^2^ Department of Nephrology and Endocrinology Copenhagen University Hospital—Rigshospitalet Copenhagen Denmark; ^3^ Department of Clinical Medicine University of Copenhagen Copenhagen Denmark; ^4^ Department of Oncology Copenhagen University Hospital—Rigshospitalet Copenhagen Denmark; ^5^ Department of Clinical Physiology and Nuclear Medicine & Cluster for Molecular Imaging Copenhagen University Hospital—Rigshospitalet Copenhagen Denmark; ^6^ Department of Surgery and Transplantation Copenhagen University Hospital—Rigshospitalet Copenhagen Denmark; ^7^ Department of Biomedical Sciences University of Copenhagen Copenhagen Denmark; ^8^ Department of Pathology Copenhagen University Hospital—Rigshospitalet Copenhagen Denmark

**Keywords:** chemotherapy, peptide receptor radionuclide therapy, small intestinal neuroendocrine neoplasm, somatostatin analogues, treatment response

## Abstract

Small intestinal neuroendocrine tumors (siNET) are rare malignancies, often diagnosed at advanced stages with metastatic spread. While surgery is the only curative treatment, medical therapies, including somatostatin analogues (SSA), peptide receptor radionuclide therapy (PRRT), and other systemic treatments, are essential for disease stabilization. The aim was to assess median progression free survival (mPFS), and prognostic factors for the most frequently used medical treatment modalities in patients with unresectable disease. It was a retrospective single‐center cohort study, including 378 patients diagnosed with siNET between 2000 and 2020. The median overall survival (mOS) for the cohort was 97 (95% CI: 83–111) months. Median PFS for octreotide and lanreotide treatment/treated patients (*n* = 255) was 30 (95% CI: 24–36) months and 5 years PFS was 32%, with no significant difference between the two agents. Risk factors for disease progression included age, Ki‐67 index, and gender (female as a protective factor). Median PFS for PRRT (*n* = 140) was 31 (95% CI: 25–37) months. Thirty‐seven patients who had PFS > 18 months after the first 4 cycles received another 2 cycles of PRRT. Median PFS after the first 4 cycles was 37 (95% CI: 30–44) months versus 10 (95% CI: 6–14) months after the 2 additional PRRT cycles. Patients treated with everolimus had a median PFS of 5 (95% CI: 0.3–10) months, and chemotherapy with streptozocin and 5‐fluorouracil resulted in a median PFS of 8 (95% CI: 5–11) months. In conclusion, SSA remains the cornerstone of first‐line therapy for unresectable siNET, with PRRT offering a valuable alternative for patients with progression on SSA. Re‐introduction of PRRT with 2 additional cycles had reduced efficacy compared with the initial treatment. PFS was short in non‐somatostatin receptor‐based therapies like everolimus and chemotherapy.

## 1 INTRODUCTION

Small intestinal neuroendocrine tumors (siNET) are rare, typically slow‐growing neoplasms with a low mitotic index. Despite their indolent growth, they are malignant, and most patients have metastases at diagnosis.^1^, ^2^, ^3^ Neuroendocrine tumors are histologically graded into three categories (G1–G3) according to the Ki‐67 proliferation index (G1, Ki‐67 <3%; G2, Ki‐67 3%–20%; G3, Ki‐67 >20%). Among the various NET entities, siNET typically exhibit low proliferative activity, and a substantial proportion are classified as G1. Another hallmark of well‐differentiated NET is the expression of somatostatin receptors, which can be demonstrated by immunohistochemistry and visualized in vivo using somatostatin receptor‐based imaging.^4^

Although the incidence of siNET remains low, improved imaging and increased clinical awareness have led to increased diagnosis.^5^, ^6^, ^7^ Disseminated disease with liver metastases is frequently accompanied by the carcinoid syndrome with flushing and diarrhea, and in severe cases carcinoid heart disease.^8^, ^9^

Surgery is the only curative treatment option and should always be considered, but the extent of metastatic spread often limits the feasibility of curative surgery.^10^, ^11^ In case of unresectable disease, somatostatin analogues (SSA) have for many years been considered as first line medical therapy for disease stabilization and hormonal control. Peptide receptor radionuclide therapy (PRRT), everolimus, interferon‐alpha (IFN‐α), and chemotherapy offer additional treatment options.^12^, ^13^, ^14^, ^15^, ^16^, ^17^

The rarity of siNET presents significant challenges in accumulating robust clinical data on treatment efficacy. Unlike more prevalent malignancies, where large randomized controlled trials guide therapeutic decisions, research on siNET primarily consists of smaller cohort studies, retrospective analyses, and expert consensus.

This study presents data from a large cohort of siNET patients who received medical treatment for unresectable disease. The primary objective was to evaluate median progression‐free survival (mPFS) linked to the most commonly used medical treatments—SSA, PRRT, everolimus, IFN‐α, and chemotherapy with a combination of streptozocin (STZ) and 5‐fluorouracil (5‐FU). The secondary outcome was to determine prognostic factors. A special focus will be on somatostatin receptor‐based therapy, which has been utilized in our institution for 25 years.

## 2 METHODS

### 2.1 Study population

The study cohort consisted of consecutive patients diagnosed with siNET who received medical treatment with an antiproliferative intent at ENETS center of Excellence, Rigshospitalet, Copenhagen. Patients diagnosed between January 1, 2000, and December 30, 2020, were included. The cohort included all patients (G1–G3) with unresectable disease (at baseline or unresectable disease during follow‐up after surgery), remnant tumor after resection, patients with potential resectable disease but not candidates for surgery, or patients who refused surgery. The total number of patients treated in our institution during the study period was 615. No patients receiving medical treatment with an antiproliferative intent were excluded. To ensure robust statistical analysis, only medical treatments administered to at least 20 patients in the cohort were included in the evaluation of treatment responses. Tumor grade was determined through immunohistochemistry of biopsy or resected tissue, based on the proliferation index (Ki‐67 index). Ki‐67 index was assessed on available tumor tissue, which could include specimens from either the primary tumor or metastatic lesions. When more than one specimen was available, the highest Ki‐67 index value was used for the analyses. In addition, the prognostic value of biochemical markers chromogranin A (CgA) and 5‐hydroxyindoleacetic acid (5‐HIAA) was evaluated and included in the analyses. Detailed data regarding this cohort has been published previously.^18^

### 2.2 Data acquisition

At the time of diagnosis, baseline data were prospectively collected and recorded in a dedicated database. This information included demographic details, tumor characteristics, and immunohistochemistry for Ki‐67‐index. The stage of disease at baseline was classified based on pathological findings and/or imaging and categorized into three groups: (1) local disease defined as primary tumor with or without regional lymph node metastases, (2) disseminated disease with other intra‐abdominal metastasis, and (3) disseminated disease with extra‐abdominal metastasis.

In accordance with the study protocol, follow‐up data were systematically gathered, including details on treatment modalities and PFS for each medical intervention. Due to the Danish unique national identification number system and comprehensive nationwide electronic medical record access, complete follow‐up was ensured, with no patients lost to follow up. Patients were tracked continuously until either death or the end of the study's follow‐up period on December 31, 2021.

### 2.3 Outcome

The primary outcome was mPFS related to specific treatments. The secondary outcome was prognostic factors related to mPFS. Median PFS was measured from the start of treatment to the point of progression, defined as death from any cause, clinical progression defined as a clinically meaningful deterioration in performance status attributable to siNET, or radiological progression as assessed in routine clinical practice applying routine clinical CT, with or without adjunct somatostatin receptor‐based imaging and/or MRI. Imaging was not re‐evaluated for study purposes. Treatments administered solely for purposes other than tumor control, such as continuous use of somatostatin analogues to manage hormonal excess after progression, were excluded from statistical analyses of PFS. Overall disease‐specific survival and median recurrence‐free survival following intended radical surgery for this cohort have been published previously.^18^

### 2.4 Statistics

Descriptive statistics were used to summarize demographic and clinical characteristics, with categorical variables presented as frequencies and percentages, and continuous variables as means with standard deviation (SD) or medians with interquartile range (IQR), when appropriate. Log2 transformation was used to approximate a normal distribution for plasma chromogranin A (p‐CgA), Ki‐67 index, plasma, and urine 5‐Hydroxyindoleacetic acid (5‐HIAA).

Univariable Cox regression analyses were performed to investigate potential associations with mPFS, including the following variables: gender, age at diagnosis, year of diagnosis (2000–2009 vs. 2010–2020), Log2(p‐CgA), Log2(Ki‐67‐index), Log2(Urine‐5‐HIAA), Log2(Plasma‐5‐HIAA), disease stage at baseline, primary surgical intervention, treatment line (first‐line vs. subsequent lines), and for the group receiving SSA: subtype of SSA (octreotide or lanreotide). Variables with a *p*‐value ≤.2 were incorporated into multivariable Coxregression analyses using backward elimination (conditional). Age and gender were included in all multivariable models. Results are presented as hazard ratios (HR) with 95% confidence intervals and corresponding *p*‐value, with HRs for Ki‐67‐index and p‐CgA reported per two‐fold increase in the non‐log‐transformed variable.

To prevent overfitting, Coxregression analyses were restricted to treatments administered to at least 40 patients. Additionally, PFS was estimated using Kaplan–Meier curves, with group differences assessed by the log‐rank test. A *p*‐value of ≤.05 was deemed statistically significant. All analyses were conducted using IBM SPSS Statistics software (Version 28.0.0.0).

### 2.5 Ethics

The study was approved by the local data protection agency at Rigshospitalet 22 July 2011 (2007‐58‐0015) and by the Danish Patient Safety Authority 14 August 2020 (31‐1521‐453). Due to the retrospective design, informed consent was not required.

## 3 RESULTS

### 3.1 Baseline characteristics

Table 1 presents the baseline characteristics. In total, 378 patients with a mean age of 65 (±11) years were included in the cohort with a median follow‐up of 55 (29–95) months. One hundred patients (28%) were categorized as NET G1, 254 (70%) as NET G2, and 7 (2%) as NET G3. KI‐67 index was missing in the remaining 17 individuals. The median Ki‐67 index was 4 (2–8) (range 1–35)%. At the initial diagnosis, 97 patients (26%) had local disease with or without mesenteric lymph node metastasis, 213 patients (56%) had other intra‐abdominal metastasis, and 68 (18%) had extra‐abdominal metastasis at the initial diagnosis. The median urinary free 5‐Hydroxyindoleacetic acid (5‐HIAA) was 70 (34–247) umol/day (normal range <40 μmol/day), whereas median plasma 5‐HIAA was 274 (84–841) nmol/L (normal range 35–123 nmol/L).

**TABLE 1:** Characteristics.

	Valid cases (*n*)	
Age, years (mean ± SD)	378	65 (**±**11)
Gender, *n* (%); Female (%)	378	182 (48%)
Year of diagnosis	378	
2000–2010		97 (26%)
2010–2020		281 (74%)
WHO grade NET, *n* (%)	361	
NET G1		100 (28%)
NET G2		254 (70%)
NET G3		7 (2%)
Stage at diagnosis, *n* (%)	378	
Local and Local + mesenteric		97 (26%)
Intra‐abdominal		213 (56%)
Extra‐abdominal		68 (18%)
Ki‐67‐index, %, (median (IQR))^a^	361	4 (2;8)
Plasma CgA, pmol/L (median (IQR))^b^	369	770 (248–2160)
Urine 5‐HIAA, μmol/day (median (IQR))^c^	209	70 (34–247)
Plasma 5‐HIAA, nmol/L (median (IQR))^c^	15	274 (84–841)

^a^ Proliferation‐index (Ki‐67‐index).

^b^ Plasma chromogranin A (CgA). Plasma CgA normal range is 30–130 L.

^c^ 5‐Hydroxyindoleacetic acid (5‐HIAA). Urine 5‐HIAA normal range is <40 μmol/day; Plasma Urine 5‐HIAA normal range is 35–123 nmol/L.

### 3.2 Somatostatin analogues (SSA)

A total of 262 (69%) patients, age 66 (±11) years, were treated with long‐acting SSA as tumor stabilizing treatment. Ninety‐five patients were treated with octreotide (target dose 30 mg/4 week), 117 with lanreotide (target dose 120 mg/4 week), and 50 patients switched between lanreotide and octreotide during the study treatment period due to adverse effects, convenience, or issues related to drug availability. If carcinoid symptoms were not controlled, the monthly dose was increased or interval reduced. The exact average dose was not recorded. SSA was first‐line treatment to 255 patients (97%) with a median Ki‐67‐index of 4 (2;7)% (G1 74 (29%), G2 181 (71%)). Seventy‐seven (29%) patients had local disease, 133 (51%) patients had intra‐abdominal metastases and 52 (20%) patients had extra‐abdominal metastases. SSA were primarily used as first‐line therapy in the second decade of the study period: 25/97 (26%) patients diagnosed in 2000–2010 versus 230/281 (82%) patients diagnosed in 2010–2020 received an SSA as first‐line treatment.

**FIGURE 1:**
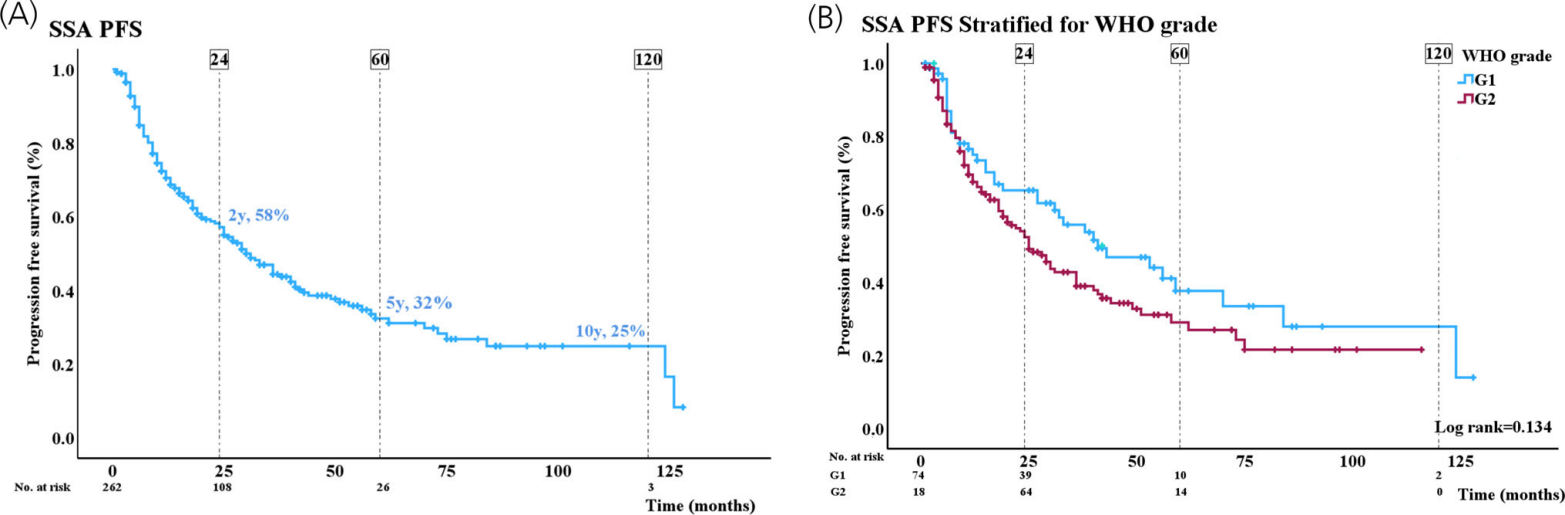
(A) Kaplan–Meier curve showing 2‐, 5‐, and 10‐year progression‐free survival (%) in patients treated with somatostatin analogues (SSA). (B) Kaplan–Meier curve showing progression‐free survival stratified by WHO grade. Differences in WHO grade were assessed by log‐rank test.

Median PFS was 30 (95% CI: 24–36) months and 5 years PFS was 32%. Overall PFS and PFS stratified for WHO grade are shown in Figure 1A,B. Risk factors for disease progression identified through uni‐ and multivariable Cox regression analysis are shown in Table 2. Independent significant risk factors for PFS were age, Ki‐67 index, gender, and primary tumor resection. Female gender and resection of primary tumor were associated with a better prognosis (Table 2). Unless patients had intolerable gastrointestinal side effects, SSA treatment was continued after disease progression to manage carcinoid symptoms and reduce risk of hormone‐related complications. No significant differences in mPFS for octreotide and lanreotide were observed with mPFS of 27 (CI: 10–44) months and 31 (CI: 6–42) months (*p* = .29), respectively.

**TABLE 2:** Prognostic factors for PFS for SSA treatment.

Variables	Univariable analysis		
	HR	95% CI	*p* value
Age (*n* = 262)	1.02	1.01–1.04	.012
Gender (*n* = 262). Ref: Female	1.30	0.92–1.83	.13
Year of diagnosis. Ref: year 2000–2010	1.47	0.86–2.52	.16
Primary surgery (*n* = 262). Ref: Surgery	2.18	1.55–3.05	<.001
Stage at diagnosis (*n* = 262). Ref: Local			<.001
Intra‐abdominal	2.19	1.42	<.001
Extra‐abdominal	2.69	1.61	<.001
WHO grade (*n* = 255)			.14
G1 vs. G2	1.40	0.96–2.04	.09
Carcinoid heart disease at diagnosis (*n* = 262). Ref: yes	0.43	0.19–0.98	.045
Ki‐67‐index (log2) (*n* = 255)^a^	1.48	1.26–1.74	<.001
Plasma CgA (Log2) (*n* = 257)^b^	1.16	1.07–1.25	<.001
Urine 5‐HIAA (Log2) (*n* = 162)^c^	1.29	1.14–1.46	<.001

Variables	Multivariable analysis		
	HR	95% CI	*p*‐value
Age	1.02	1.00–1.04	.**041**
Gender. Ref: Female	1.43	1.01–2.03	.**045**
Primary surgery. Ref: Surgery	1.86	1.28–2.68	.**001**
Ki‐67‐index (log2)	1.38	1.15–1.65	**<.001**

*Note*: Bold type represents significant (*p* < .05) results in multivariable analysis.

^a^ Proliferation‐index (Ki‐67‐index).

^b^ Plasma chromogranin A (CgA).

^c^ 5‐Hydroxyindoleacetic acid (5‐HIAA).

### 3.3 Peptide receptor radionuclide therapy (PRRT)

**FIGURE 2:**
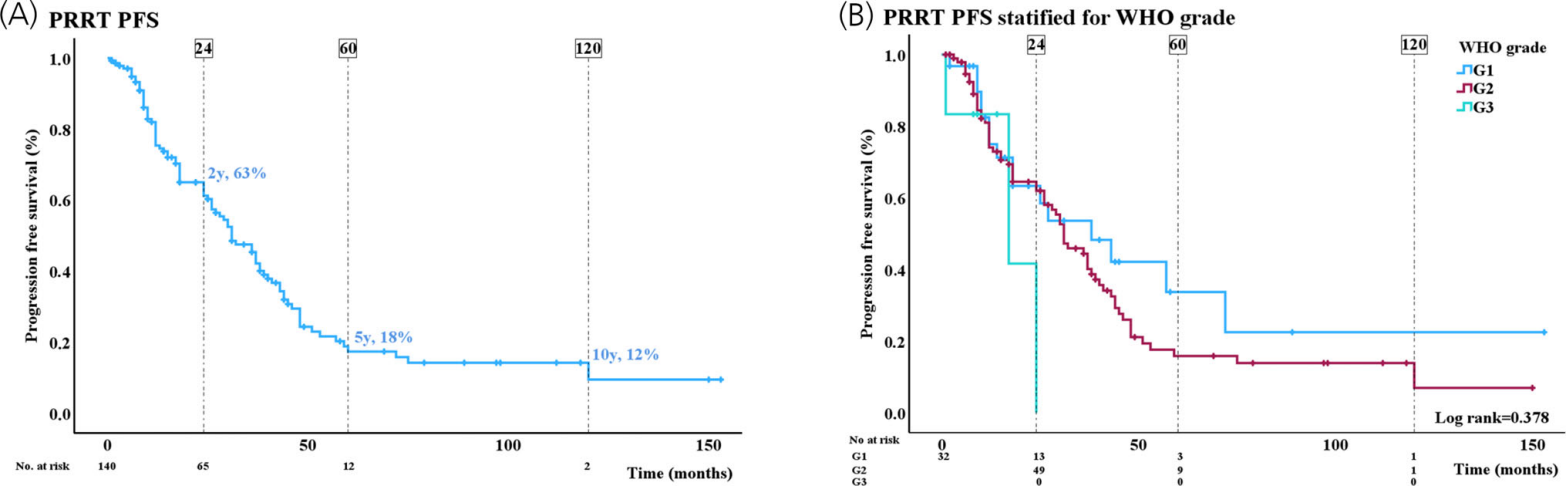
(A) Kaplan–Meier curve showing 2‐, 5‐, and 10‐year progression‐free survival (%) in patients treated with peptide receptor radionuclide therapy (PRRT). (B) Kaplan–Meier curve showing progression‐free survival stratified by WHO grade. Differences in WHO grade were assessed by a log‐rank test.

PRRT was administered in 140 patients, age 61 (±10) years, in seven patients (8%) as first‐line treatment. The median Ki‐67‐index was 5 (3;10)%, G1 32 (24%), G2 98 (72%), G3 6 (4%). Sixteen (11%) patients had local but unresectable disease, 95 (68%) patients had intra‐abdominal metastases and 29 (21%) patients had extra‐abdominal metastases. A standard treatment regimen comprising four cycles of 7.4 GBq Lu‐177‐DOTATATE with 8 weeks intervals was used. For renal protection, an amino‐acid solution was co‐administered following international guidelines. Median PFS from the first cycle of treatment to the time of progression was 31 (CI: 25–37) months. Overall PFS and PFS stratified for WHO grade are shown in Figure 2A,B. The only significant risk factor in multivariable analysis was stage at diagnosis (local disease as reference, *p* = .041; HR (intra‐abdominal): 2.08; (CI: 0.92–4.52), HR (extra‐abdominal): 3.04, (CI: 1.27–7.32)) (Table 3).

**TABLE 3:** Prognostic factors for PFS for PPRT treatment.

Variables	Univariable analysis		
	HR	95% CI	*p*‐value
Age (*n* = 140)	1.02	1.00–1.04	.13
Stage (*n* = 140), Ref: Local			.026
Intra‐abdominal metastasis	2.08	0.94–4.57	.07
Extra‐abdominal metastasis	3.22	1.35–7.70	.009
Line of treatment (*n* = 140), first vs. second or more.			
Ref: first‐line	2.05	0.75–5.62	.16
Carcinoid heart disease at diagnosis (*n* = 140). Ref: yes	0.46	0.15–1.48	.19
Ki‐67‐index (log2) (*n* = 136)^a^	1.15	0.97–1.37	.10
Plasma CgA (log2) (*n* = 138)^b^	1.08	0.99–1.18	.09

Variables	Multivariable analysis		
	HR	95% CI	*p*‐value
Stage Ref: Local			.**041**
Intra‐abdominal metastasis	2.04	0.92–4.52	.077
Extra‐abdominal metastasis	3.04	1.27–7.32	.**013**

*Note*: Bold type represents significant (**p < .05) results in multivariable analysis.

^a^ Proliferation‐index (Ki‐67‐index).

^b^ Plasma chromogranin A (CgA).

Six patients with NET G3 were treated with PRRT. PFS following PRRT ranged from 1 to 24 months:

Patient 1: Ki‐67‐index 21%, PRRT as third‐line therapy, PFS 1 month, prior treatment everolimus and temozolomide.
Patient 2: Ki67‐index 25%, PRRT as third‐line, PFS 8 months, prior treatment etoposide and temozolomide/capecitabine combination.
Patient 3: Ki‐67 index of 35%, PRRT as third line, PFS 9 months, prior treatment SSA and temozolomide/capecitabine combination.
Patient 4: Ki‐67‐index of 22%, PRRT as second‐line, PFS 14 months, prior treatment temozolomid.
Patient 5: Ki‐67‐index 20%, PRRT third‐line, PFS 17 months, prior treatment etoposide and a combination of streptozocin (STZ) and 5‐fluorouracil (5‐FU).
Patient 6: Ki‐67‐index 27%, PRRT third‐line, PFS 24 months, prior treatment SSA and temozolomid/capecitabin combination.

**FIGURE 3:**
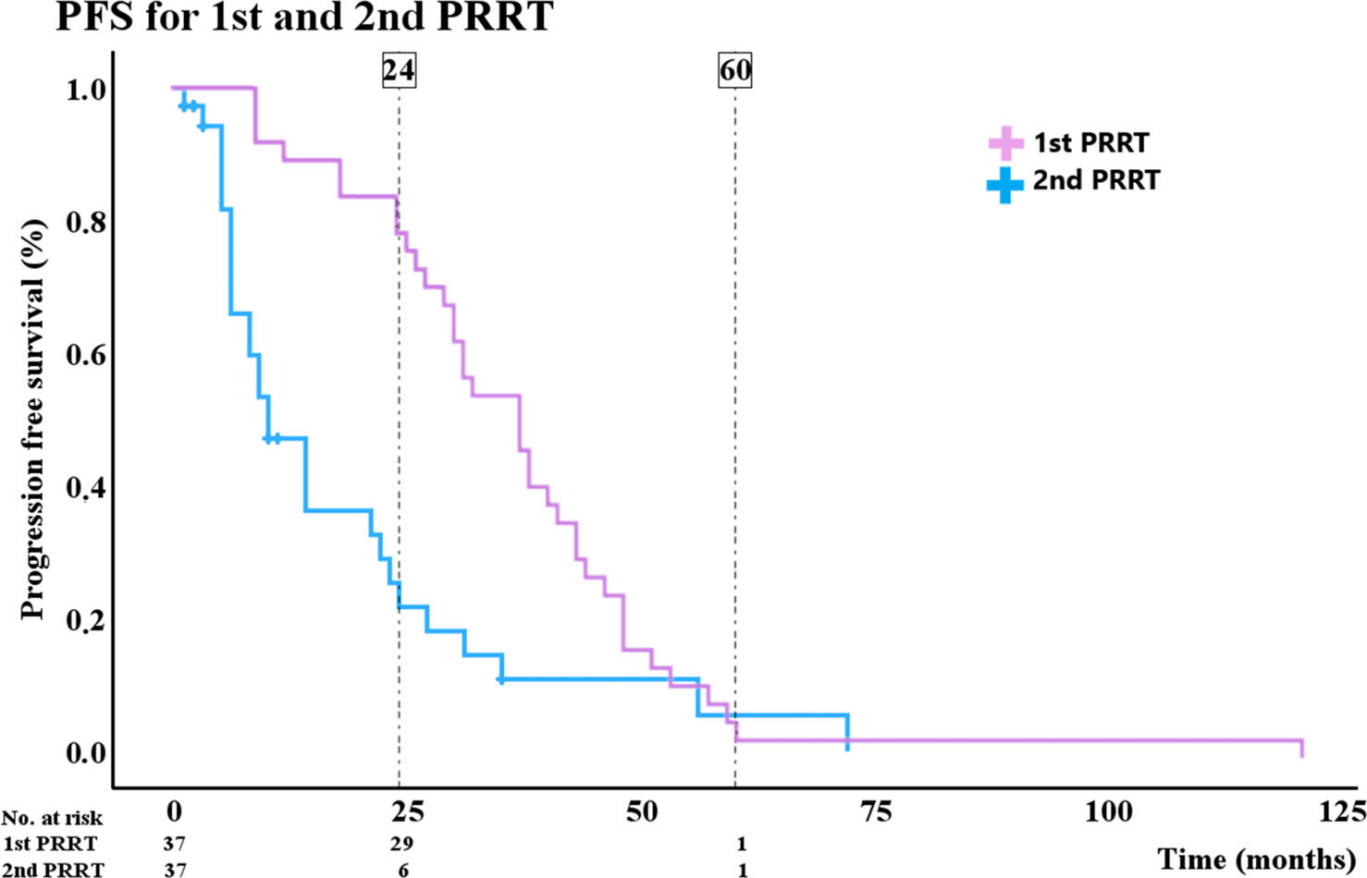
Kaplan–Meier curve showing 2‐ and 5‐year progression‐free survival (%) in a group of patients (*n* = 37) re‐treated with 2 extra cycles of peptide receptor radionuclide therapy (PRRT). The figure shows the difference in progression‐free survival in the first cycle of treatment vs. the second cycle of treatment.

Thirty‐seven patients, age 57 (±10) years, who had a good response to the initial four cycles of PRRT (PFS >18 months) received a second PRRT comprising 2 cycles of PRRT 7.4 GBq. The median Ki‐67 index was 6 (4–8)%, G1: 6 (17%), G2: 28 (80%), G3: 1 (3%). Three (8%) patients had local disease, 24 (65%) patients had intra‐abdominal metastases and 10 (27%) patients had extra‐abdominal metastases. Median PFS after the first 4 cycles was 37 (CI: 30–44) months versus 10 (CI: 6–14) months after the 2 additional PRRT cycles (Figure 3).

### 3.4 IFN‐α

A total of 71 patients, age 63 (±10) years, received IFN‐α. Seventy % received IFN‐α as other than first‐line therapy. The median Ki‐67‐index was 4 (2–8)%, G1 23 (35%), G2 42 (65%). Twelve (17%) patients had local disease, 45 (63%) patients had intra‐abdominal metastases and 14 (20%) patients had extra‐abdominal metastases. IFN‐α was primarily used in the first decade of the study period; 37/97 (38%) patients diagnosed in 2000–2010 versus 34/281 (12%) patients diagnosed in 2010–2020 were treated with IFN‐α.

The median PFS was 18 (CI: 14–22) months. Independent significant risk factors were age, p‐CgA, gender, year of diagnosis, and stage at diagnosis. Female and treatment in the first decade (2000–2009) were associated with a better prognosis (Table 4).

**TABLE 4:** Prognostic factors for PFS for interferon‐alpha treatment.

Variables	Univariable analysis		
	HR	95% CI	*p*‐value
Age (*n* = 71)	1.02	1.00–1.052	.081
Year at diagnosis (*n* = 71)	2.10	1.20–3.66	.009
Stage (*n* = 71). Ref: Local			.19
Intra‐abdominal metastasis	1.22	0.60–2.50	.58
Extra‐abdominal metastasis	2.09	0.87–5.02	.097
Ki‐67‐index (Log2) (*n* = 65)^a^	1.36	1.04–1.79	.026
Plasma CgA (Log2) (*n* = 69)^b^	1.14	1.03–1.27	.015
Urine 5‐HIAA (Log2) (*n* = 31)^c^	1.33	1.04–1.70	.022

Variables	Multivariable analysis		
	HR	95% CI	*p*‐value
Age	0.17	0.05–0.59	.**006**
Gender. Ref: Female	1.10	1.02–1.19	.**014**
Year at diagnosis	14.19	2.75–73.15	.**002**
Stage Ref: Local			.**038**
Intra‐abdominal metastasis	0.23	0.04–1.23	.085
Extra‐abdominal metastasis	0.04	0.003–0.51	.**013**
Plasma CgA (Log2)	1.28	1.03–1.58	.**025**

*Note*: Bold type represents significant (*p* < .05) results in multivariable analysis.

^a^ Proliferation‐index (Ki‐67‐index).

^b^ Plasma chromogranin A (CgA).

^c^ 5‐Hydroxyindoleacetic acid (5‐HIAA).

### 3.5 Streptozocin and 5‐fluorouracil (STZ/5‐FU)

A total of 48 patients, age 63 (±10) years, received chemotherapy with a combination of streptozocin (STZ) and 5‐fluorouracil (5‐FU). The induction dose of STZ was 500 mg/m^2^ on day 1–5, combined with 5‐FU 400 mg/m^2^ on day 1–3 first week, followed by STZ 1000 mg/m^2^ and 5‐FU at 400 mg/m^2^ every 3rd week.

STZ/5‐FU was used as first‐line therapy in 23/48 (48%) patients and as for IFN‐α, chemotherapy was used more frequently in the first decade of the study period (28/97 (29%) patients were diagnosed in 2000–2010 vs. 20/281 (7%) patients in 2010–2020).

The median Ki‐67‐index was 10 (5–15)%, G1: 5 (12%), G2: 34 (81%), and G3: 3 (7%). Five (10%) patients had local disease, and 34 (71%) and 9 (19%) patients had intra‐abdominal and extra‐abdominal metastases, respectively. Median PFS was 8 (CI: 5–11) months. U‐5‐HIAA was the only significant independent prognostic factor (Table 5).

**TABLE 5:** Prognostic factors for PFS for streptozocin and 5‐fluorouracil treatment.

Variables	Univariable analysis		
	HR	95% CI	*p*‐value
Gender (*n* = 48). Ref: Female	1.60	0.83–3.10	.16
Ki‐67‐index (log2) (*n* = 42)^a^	1.25	0.90–1.73	.18
Plasma CgA (log2) (*n* = 47)^b^	1.12	0.99–1.26	.08
Urine 5‐HIAA (log2) (*n* = 26)^c^	1.30	1.03–1.64	.026

Variables	Multivariable analysis incl. Ki‐67‐index		
	HR	95% CI	*p*‐value
Urine 5‐HIAA (Log2)	1.29	1.01–1.65	.**045**

*Note*: Bold type represents significant (*p* < .05) results in multivariable analysis.

^a^ Proliferation‐index (Ki‐67‐index).

^b^ Plasma chromogranin A (CgA).

^c^ 5‐Hydroxyindoleacetic acid (5‐HIAA).

### 3.6 Everolimus

A total of 28 patients received everolimus. Mean age was 62 (±10), and median Ki‐67‐index was 7 (3–12)%. Median PFS was 5 (CI: 0.3–10) months. Due to the low number of patients, no additional statistical analyses were performed.

Among the treatments described, SSA, PRRT, everolimus, and interferon‐alpha are approved by the European medical agency (EMA). By contrast, streptozotocin and 5‐fluorouracil chemotherapy are authorized only for pancreatic NET.

### 3.7 Overall survival

One‐hundred and eighty patients died during follow‐up. The median OS was 97 (CI: 83–111) months with a 2‐, 5‐, and 10‐year survival of 85%, 68%, and 40%, respectively. Median OS stratified for WHO grade was 112 (92;132) months for G1, 96 (76;116) months for G2, and 43 months for G3.

### 3.8 Post hoc analyses

**FIGURE 4:**
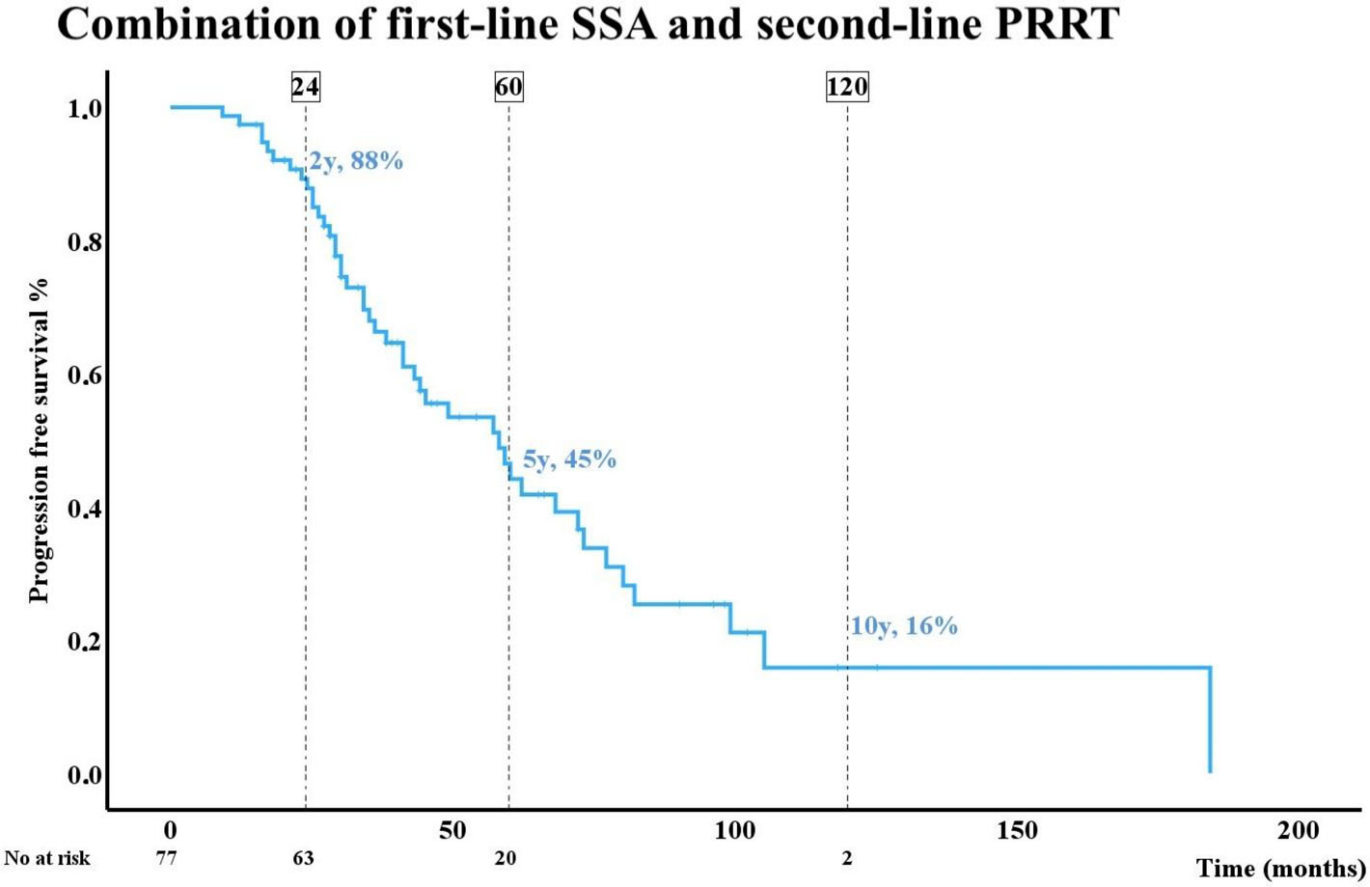
Kaplan–Meier curve showing the 2‐, 5‐, and 10‐years progression‐free survival (%) for the combination of somatostatin analogues (SSA) as first‐line treatment followed by radionuclide therapy (PRRT) as second‐line.

SSA are generally accepted as first‐line treatment for siNET, whereas the optimal choice of second‐line therapy remains debated. This prompted us to examine the combined PFS associated with first‐line SSA followed by relevant second‐line treatments; the treatment sequences are summarized in Table 6. As shown, only the SSA plus PRRT sequence was supported by sufficient data to permit formal analyses. The results are presented in Figure 4, demonstrating a 5‐year PFS of 45% for the SSA‐to‐PRRT treatment sequence.

**TABLE 6:** Combination of first‐line SSA and other treatment modalities as second‐line treatment.

Second‐line treatment	Age (mean ± SD)	Ki‐67‐index, % (median (IQR))
PRRT^a^ (*n* = 77)	61.6 ± 11	6 (3;9)
Interferon‐alpha (*n* = 31)	62.8 ± 9	4 (2;6)
Streptozocin and 5‐fluorouracil (*n* = 10)	64.1 ± 11	15 (10;15)
Everolimus (*n* = 3)	67.3 ± 8	4 (1;4;13)^b^

^a^ Peptide receptor radionuclide therapy.

^b^ Ki‐67‐index of all three patients in this group is shown.

## 4 DISCUSSION

The study showed that treatment with SSA and PRRT aligned with results obtained in randomized studies, and that re‐introduction of PRRT with 2 additional cycles was inferior compared with the initial treatment. Non‐somatostatin receptor‐based therapies showed limited efficacy.

SSA, primarily targeting somatostatin receptor type 2—octreotide and lanreotide—have passed the test of time and are well established as first‐line treatment of unresectable siNET.^11^, ^19^, ^20^ The efficacy of SSA has been documented in the PROMID Study (total *n* = 85 from 2009^13^) showing mPFS of 14 months in patients treated with long acting octreotide versus 6 months in controls and in the CLARINET study from 2014^12^ utilizing lanreotide autogel (total numbers of siNET *n* = 73). Median PFS was 18 months in the controls and not met in the treatment arm, where ~40% had recurrence after 27 months. Despite the difference in mPFS, which might reflect differences in selected patient groups, the two compounds are considered equivalent in various guidelines. The present study includes to our knowledge the largest cohort treated with SSA in siNET. Median PFS of 30 months was substantially longer than reported in the PROMID Study but somewhat similar to what was reported in the CLARINET study and in agreement with other retrospective studies.^21^ A recently published Scandinavian study, which only included siNET G2 patients, found a mPFS of 31 months among a subgroup of patients with a Ki‐67‐index 3%–5%, which is similar to our results.^14^ As independent risk factors for progression we identified age and proliferation based on Ki‐67‐index expressed by grade. Thus, our data supports that proliferation has an impact on mPFS in patients on SSA treatment which is in agreement with previous studies.^14^ However, there was no difference in mPFS between our patients treated with either octreotide or lanreotide, which is in accordance with previous retrospective studies.^22^, ^23^ At our institution, the selection between octreotide and lanreotide is primarily guided by pricing established through annual tender procedures, which minimizes the risk of selection bias affecting our results.

Most cases of progression in our cohort occurred while patients were treated with maximum recommended dose (octreotide 30 mg every 4th week/lanreotide 120 mg every 4th week). Few patients were on high dose treatment before progression to reduce hormone related symptoms. After radiological progression we often increased the dose, but the precise doses and PFS after dose escalation were not routinely registered. Prospective data on dose escalation are scarce. The single‐arm phase II CLARINET‐FORTE study showed a median PFS of 8.3 months after step‐wise increase of lanreotide every 14 days to 120 mg.^24^ An Italian multicenter retrospective study found a median PFS of 31 months using heterogeneous high‐dose or shortened‐interval regimens.^25^ Collectively, these observations suggest that dose escalation can prolong disease control in selected patients, but they underscore the need for systematic dose documentation and prospective randomized trials.

PRRT has been used in Europe for more than 15 years for patients with well‐differentiated G1–G2 NET progressing on SSA. Its efficacy and tolerability were initially supported by multiple retrospective studies and later confirmed in a randomized controlled trial published in 2017 (NETTER‐1).^15^ In the final analysis, patients treated with ^177^Lu‐DOTATATE had a mPFS of 28.4 months, compared with 8.5 months in the high‐dose octreotide LAR control arm.^26^ These findings are consistent with our own data and with results from large retrospective cohorts, including a Dutch series of 443 patients,^27^ and a recent review summarizing outcomes across multiple studies.^28^ In our cohort, six patients with NET G3 received PRRT. Owing to the small sample size, formal statistical analyses were not feasible; however, several of these patients appeared to derive clinical benefit. This is in line with previous reports indicating a median PFS of ~14 months.^29^

It is widely accepted and approved by EMA and FDA that initial PRRT consists of four treatment cycles of 7.4 GBq administered at ~8‐week intervals. However, the optimal approach to renewed treatment following a favorable initial response remains poorly defined. We routinely administered two additional ^177^Lu‐DOTATATE cycles to patients who had disease control for more than 12 months after the initial four‐cycle course, a strategy already described in salvage series that applied the same two‐cycle retreatment after a durable first response of 12–18 months.^30^, ^31^ The findings from other studies employing two additional PRRT cycles are consistent with our results, demonstrating substantially reduced efficacy compared with the initial four‐cycle treatment course.^30^, ^32^ While two additional cycles remain common practice, several alternative strategies have been proposed. A French randomized phase II study (ReLUTH) is currently evaluating four versus two additional cycles of ^177^Lu‐DOTATATE in well‐differentiated small‐intestinal NET.^33^ Another approach is dosimetry‐guided PRRT, aiming to deliver the maximum tolerated absorbed dose to the kidneys (up to 23 Gy) during 4 treatment cycles.^34^ Finally, targeted α‐therapy with, for example, ^225^Ac‐DOTATATE is emerging as a potential future salvage option after maximal β‐emitter exposure.^35^, ^36^ Together, these data illustrate that retreatment need not be limited to two cycles and that personalized approaches warrant further prospective evaluation.

A total of 71 patients received IFN‐α, most often not as first‐line therapy and frequently in combination with SSA. The clinical use of IFN‐α is limited by its availability and significant side effects. In our cohort, the observed treatment effect was modest and in line with findings from previous retrospective series. Notably, IFN‐α has never been investigated in a randomized study in patients with siNET, and evidence supporting its use is primarily based on older observational studies and clinical experience.^37^

Chemotherapy with STZ/5‐FU has been used in the treatment of well‐differentiated NET since the 1970s, and is still recommended in the 2024 ENETS guidelines for selected cases, particularly those with a Ki‐67‐index >15% or exhibiting rapid tumor progression despite its limited efficacy in this patient group.^20^, ^38^ In our study, which comprises a relatively large series of siNET patients treated with STZ/5‐FU, we found a short mPFS of 8 months; half of the patients received the regimen as first‐line therapy. Over the past decade, we have used STZ/5‐FU only rarely, and exclusively in highly selected patients with high‐grade G2 or G3 disease.

Everolimus is approved for the treatment of advanced NET including siNET based on the RADIANT‐4 trial, which demonstrated a mPFS of 11.0 months compared with 3.9 months in the placebo arm.^17^ In our cohort, however, the efficacy of everolimus appeared limited, with a median PFS of only 5 months. This likely reflects both intrinsic resistance mechanisms and the fact that everolimus is often used as a late‐line treatment option in our center, typically in patients with declining performance status and limited remaining options. Comparative data between everolimus and other treatment modalities remain limited. Notably, at the ENETS meeting in March 2025, results from the phase III COMPETE trial were presented, showing a mPFS of 23.9 months for PRRT versus 14.1 months for everolimus in patients with well‐differentiated, progressive GEP‐NET.^39^

Our data reflect a clear temporal shift in treatment selection over the 2000–2020 period. In the most recent decade, first‐line therapy was dominated by SSA, with peptide receptor radionuclide therapy (PRRT) commonly used as second‐line treatment. In contrast, during the early 2000s, patient numbers were lower and systemic treatment more often relied on chemotherapy and interferon‐alpha. To date, the optimal choice of second therapy in siNET remains debated. Within the current treatment landscape, the most relevant choice for patients with well‐differentiated siNET G1–G2 is between PRRT and everolimus. At our institution, PRRT is considered the preferred second option, and everolimus is only used when somatostatin receptor‐based imaging demonstrates low uptake and/or when impaired renal function limits the feasibility of PRRT. Consequently, our data do not allow a direct comparison between everolimus versus PRRT as second line treatment. In our cohort, the combined PFS associated with somatostatin receptor–based therapy approached 5 years. The COMPETE trial, reporting improved PFS with PRRT compared with everolimus, is further supported by real‐world evidence indicating substantially longer PFS with PRRT compared with both targeted therapies and chemotherapy.^40^

This study benefits from a substantial sample size and highly detailed clinical data obtained directly from patient records, with complete follow‐up for all individuals. Nevertheless, several limitations should be acknowledged. The retrospective design may be associated with incomplete data capture, and evolving treatment guidelines over the study period have influenced both treatment sequencing and the availability of therapeutic options. Ideally data capture should not have stopped in 2020. Patient classification was based on diagnostic data, but for some individuals, disease progression later warranted an update of tumor grading. This may have introduced discrepancies in WHO grade assignment at the time of subsequent therapies. Furthermore, as most patients received multiple lines of treatment, there is considerable overlap between treatment groups, which may complicate interpretation of treatment‐specific outcomes. Furthermore, the vast majority of patients continued SSA therapy after disease progression; consequently, the observed efficacy of subsequent treatment modalities cannot be interpreted as monotherapy effects. However, continuation of SSA is recommended to maintain control of hormone‐related symptoms, and thus our data reflect everyday clinical practice in accordance with current guidelines.

In conclusion, this large single center study highlights the treatment outcomes in a real world setting. The study supports the important role of somatostatin‐based therapies in the management of advanced siNET. PRRT is a valuable option for patients who progress on SSA treatment. However, re‐introduction of PRRT using two additional cycles showed reduced effectiveness compared with the initial treatment. Non‐somatostatin receptor‐based therapy, such as everolimus and chemotherapy, was administered in later stages of disease and were associated with short PFS.

## CONFLICT OF INTEREST STATEMENT

The authors declare no conflicts of interest.

## Data Availability

The data that support the findings of this study are available on request from the corresponding author. The data are not publicly available due to privacy or ethical restrictions.
